# COVID-19 Infection Deteriorates the Clinical Condition and Outcomes of Acute Pancreatitis: A Meta-Analysis

**DOI:** 10.1155/2022/6823866

**Published:** 2022-10-28

**Authors:** Yulin Guo, Shun Hu, Xiaohui Wang, Zhe Jiang, Shuangni Duan, Feng Cao, Fei Li

**Affiliations:** ^1^Department of General Surgery, Xuanwu Hospital, Capital Medical University, Beijing 100053, China; ^2^School of Public Health, Xiangnan University, Chenzhou 423000, Hunan Province, China

## Abstract

**Backgrounds:**

The novel coronavirus disease 2019 (COVID-19) has caused a global pandemic. Pancreatic injuries have been reported in COVID-19 patients. The present meta-analysis was conducted to compare the morbidity and outcomes of AP between COVID-19 positive and negative patients.

**Methods:**

Databases including Cochrane Library, PubMed, and EMBASE were systematically searched (until July 3rd 2022). Studies with English abstracts comparing the severity and outcomes of AP between COVID-19 positive and negative patients were included. Mean differences or odds ratios with a 95% confidence interval were employed for assess variables. Risk of publication bias was assessed with funnel plots.

**Results:**

Data from 7 studies with a total of 2816 AP patients were included. COVID-19 positive was associated with higher incidences of pancreatic necrosis (OR = 1.65; 95% CI: 1.13 to 2.42, *P* = 0.01; *P* = 0.82 for heterogeneity) and persistent organ failure (OR = 6.87; 95% CI: 2.37 to 19.98, *P* = 0.0004; *P* = 0.12 for heterogeneity), especially cardiovascular failure (OR = 2.92; 95% CI: 1.66 to 5.14, *P* = 0.0002; *P* = 0.58 for heterogeneity) and acute respiratory distress syndrome (ARDS) or acute lung injury (ALI) (OR = 3.03; 95% CI: 2.09 to 4.39, *P* < 0.00001; *P* = 0.20 for heterogeneity). COVID-19 infection induced a higher level of CRP (MD = 0.40; 95% CI: 0.16 to 0.64, *P* = 0.001; *P* < 0.00001 for heterogeneity) as well as coagulation disorders involving platelets, prothrombin time, activated partial thromboplastin time, and D-dimer (all *P* < 0.05). During hospitalization, COVID-19 positive was associated with higher ICU admission rate (OR = 2.76; 95% CI: 1.98 to 3.85 *P* < 0.00001; *P* = 0.47 for heterogeneity). COVID-19 positive AP was associated with a higher mortality rate (OR = 3.70; 95% CI: 2.60 to 5.25, *P* < 0.00001; *P* = 0.12 for heterogeneity). *Discussion*. The number of included studies is limited and none is RCT, thus the risks of publication and selective bias could not be ignored. COVID-19 deteriorated the severity and clinical outcomes of AP, with a high incidence of morbidity and mortality.

## 1. Introduction

The novel coronavirus disease 2019 (COVID-19) has caused a global pandemic. COVID-1 is mainly characterized by fever and respiratory symptoms, with dyspnea and lung infiltrates [1]. Apart from the respiratory symptoms, gastrointestinal symptoms have been found presented in about 20.3% of patients, part of whom were diagnosed with acute pancreatitis [2, 3]. This is due to the findings that the pancreas is the potential target organ of COVID-19, with acinar and islet cells containing ACE2 receptors [4, 5].

COVID-19 infection may contribute to AP or aggravated inflammatory response, which causes the increased risk of organ failure and complications [6, 7]. AP is a common emergency disease, and about 20–30% patients develop into severe acute pancreatitis (SAP) [8]. SAP is a life-threatening condition with the mortality rate ranging from 15% to 30% [9]. However, due to the limited published studies, the morbidity and mortality for COVID-19 patients with AP were largely undefined. Thus, the present study was conducted to compare the disease severity and outcomes of AP between COVID-19 positive and negative patients. The present study was conducted and organized in accordance with the PRISMA Checklist (Table S1).

## 2. Material and Methods

### 2.1. Search Strategy for Studies

Two authors performed comprehensive search in databases including PubMed, Cochrane Library, and EMBASE, independently. The key words and terms for the comprehensive search were (acute pancreatitis) and (COVID-19 or (SARS-CoV 2 or coronavirus)). All articles published until July 3^rd^, 2022 were retrieved to identify eligible articles comparing the disease severity and outcomes of AP between COVID-19 positive and negative patients. Moreover, a manual search was carried out on the referenced articles and related articles by supplying the article sources.

### 2.2. Inclusion and Exclusion Criteria

The following inclusion criteria should be met (1) Studies that compare the disease severity and clinical outcomes of AP between COVID-19 positive and negative patients. (2) When the eligible studies involved overlapped patients, only the better designed study could be included. (3) At least one outcome of interest should be reported, regarding disease severity, clinical outcomes, morbidity, and mortality. (4) Full text with a full English abstract should be available.

The studies which meet the following aspects should be excluded: (1) Unpublished studies, or studies presented only with an abstract. (2) Case report or case series. (3) Letter, announcement, conference, or review.

Two authors independently assessed the titles, abstracts, and full texts of retrieved studies to find potential studies referring to these criteria. EndNote X6 software was used in the study selection process. Whenever discrepancies were encountered, discussions would be held.

### 2.3. Data Extraction and Methodology Quality Assessment

Two authors performed data extraction in an independent manner. General characteristics and demographic data extracted included the following: first author and publication time, the sample size of patients, clinical characteristics on admission, and study type. Outcomes of interest included disease severity, clinical outcomes, morbidity, and mortality.

The quality assessment was carried out using the Newcastle-Ottawa Scale (NOS) for the included cohort studies. These included were scored referring to the following items: selection of patients, comparability between the groups, and assessment of outcome. A study with a score no less than six was regarded as moderate to high quality. Whenever discrepancies were encountered, discussions would be held.

### 2.4. Statistical Analysis and Calculation

All data were statistically pooled with review manager (Version 5.3, Cochrane Collaboration, Oxford, UK). Mean differences (MDs) with a 95% confidence interval (CI) and odds ratios (ORs) with 95% CI were employed to assessing continuous variables and dichotomous variables, respectively. When continuous variables were presented as median with range, the statistical method reported by Hozo et al. [10] was adopted. Heterogeneity across studies was evaluated by *I*^2^. When heterogeneity was indicated with *I*^2^ ≥ 50% or *P* < 0.1, a random effects model should be used. On the contrary, a fixed effect model would be used in the homogeneity condition. Sensitivity analysis was carried out through removing one study in each step to assess the source of heterogeneity and the stability of the pooled results. The assessment of the risk of publication bias was performed with funnel plots. Outcomes of interest were synthesized based on the items reported by each included study. A two-tailed *P* value less than 0.05 indicated statistical significance.

## 3. Results

### 3.1. Search Results, Characteristics, and Quality Assessment of Studies

A total of 409 studies were retrieved. 29 duplicates were removed. Then, after reviewing the titles and abstracts, 191 irrelevant studies, 6 letters, 68 case reports, 3 case series, and 27 reviews and comments were removed. Full texts of the remaining 17 studies were carefully read according to the inclusion and exclusion criteria. Finally, seven studies were included in the qualitative synthesis [11–17].

A total of 2816 AP patients were involved, with 438 patients in the COVID-19 positive group and 2378 patients in the COVID-19 negative group. The general characteristics of each included study were shown in Table 1. According to the Newcastle-Ottawa Scale (NOS) assessment, all included studies scored no less than six stars (Table 1). Thus, all included are of moderate to high quality.

### 3.2. The Pooled Results of Complications of AP

The pancreatic necrosis was reported by four studies, with 256 patients in the COVID-19 positive group and 1689 patients in the COVID-19 negative group [11, 12, 14, 15]. The incidence of pancreatic necrosis in the COVID-19 positive group was significantly higher than that of the COVID-19 negative group (OR = 1.65; 95% CI: 1.13 to 2.42, *P* = 0.01; *P* = 0.82 for heterogeneity) (Table 2). Portal venous thrombosis was reported by two studies, including 118 patients in the COVID-19 positive group and 1276 patients in the COVID-19 negative group [11, 14]. The incidence of portal venous thrombosis in the COVID-19 positive group was comparable to that of the COVID-19 negative group (OR = 1.02; 95% CI: 0.35 to 2.94, *P* = 0.97; *P* = 0.95 for heterogeneity) (Table 2).

Considering the data on systemic complications, persistent organ failure was reported by two studies, involving 1667 patients [11, 14]. The incidence of persistent organ failure in the COVID-19 positive group was significantly higher than that of the COVID-19 negative group (OR = 6.87; 95% CI: 2.37 to 19.89, *P* = 0.0004; *P* = 0.12 for heterogeneity) (Table 2). Furthermore, specified organ failures were analyzed. Cardiovascular failures were reported by two studies, involving 370 patients [16,17]. The incidence of cardiovascular failure in the COVID-19 positive group was significantly higher than that of the COVID-19 negative group (OR = 2.92; 95% CI: 1.66 to 5.14, *P* = 0.0002; *P* = 0.58 for heterogeneity) (Table 2). Moreover, acute respiratory distress syndrome (ARDS) or acute lung injury (ALI) was reported by four studies, with 260 patients in the COVID-19 positive group and 1712 patients in the COVID-19 negative group [11, 14, 16, 17]. The pooled results showed that the incidence of ALI/ARDS in the COVID-19 positive group was significantly higher than that of the COVID-19 negative group (OR = 3.03; 95% CI: 2.09 to 4.39, *P* < 0.00001; *P* = 0.20 for heterogeneity) (Table 2). However, the incidence of renal failure [14, 16, 17] and multiple organ dysfunction syndrome (MODS) [14, 16] in the COVID-19 positive group was comparable to those of the COVID-19 negative group (Table 2).

### 3.3. Pooled Results of Treatments and Clinical Outcomes

Regarding the treatment and clinical outcomes between the two groups, mechanical ventilation was reported by three studies, involving 579 patients [12, 14, 16]. Mechanical ventilation was performed more common in the COVID-19 positive group than the COVID-19 negative group (OR = 7.36; 95% CI: 4.19 to 12.92, *P* < 0.00001; *P* = 0.66 for heterogeneity) (Table 2). Data on noninvasive ventilation were reported by two studies, with 99 patients in the COVID-19 positive group and 291 patients in the COVID-19 negative group [14, 16]. There was no significant difference between the two groups on noninvasive ventilation (Table 2). For surgical treatment of AP, three studies with 2254 were involved [11, 15, 16]. However, no significant difference was found in the surgical rate between the COVID-19 positive and negative groups (Table 2). Data on ICU admission rate were reported by four studies, with 359 patients in the COVID-19 positive group and 1965 patients in the COVID-19 negative group [11, 13, 15, 16]. The ICU admission rate in the COVID-19 positive group was significantly higher than that of the negative group (OR = 2.76; 95% CI: 1.98 to 3.85, *P* < 0.00001; *P* = 0.47 for heterogeneity) (Table 2). Length of hospital stay was reported by four studies, with 232 patients in the COVID-19 positive group and 1789 patients in the COVID-19 negative group [11, 12, 14, 16] The length of hospital stay in the COVID-19 positive group was significantly longer than that in the COVID-19 negative group (MD = 4.53; 95% CI: 0.96 to 8.10, *P* = 0.01; *P* = 0.02 to heterogeneity). As for the mortality comparison between the two groups, data on mortality were reported by seven studies, with 406 patients in the COVID-19 positive group and 2112 patients in the COVID-19 negative group [11–17]. The mortality rate in the COVID-19 positive group was significantly higher than that of the negative group (OR = 3.70; 95% CI: 2.60 to 5.25, *P* < 0.00001; *P* = 0.12 for heterogeneity) (Table 2).

### 3.4. Pooled Results of Laboratory Tests

Apart from the clinical complications and treatment outcomes, we analyzed the laboratory results. The counts of leucocyte, lymphocyte, and platelets were reported by three studies, with 158 patients in the COVID-19 positive group and 302 in the COVID-19 negative group [13, 15, 17]. The pooled results showed that the level of lymphocyte was significantly lower in the COVID-19 positive group than the COVID-19 negative group (MD = −0.49; 95% CI: −0.74 to −0.24, *P* = 0.0001; *P* = 0.07 for heterogeneity) (Table 3), while the level of platelets was significantly higher in the COVID-19 positive group (MD = 13.41; 95% CI: 4.64 to 22.18, *P* = 0.003; *P* = 0.37 for heterogeneity) (Table 3). However, the count of leucocyte was comparable between the two groups. The coagulation functions were also analyzed, including prothrombin time and activated partial thromboplastin time (APTT), with 75 patients in the COVID-19 positive group and 196 in the COVID-19 negative group [13, 17]. Both the level of prothrombin time (MD = 0.31; 95% CI: 0.12 to 0.50, *P* = 0.001; *P* = 0.23 for heterogeneity) and APTT (MD = 1.95; 95% CI: 1.30 to 2.59, *P* < 0.00001; *P* = 0.57 for heterogeneity) were significantly lower in the COVID-19 positive group than the COVID-19 negative group (Table 3). D-dimer was reported by three studies, involving 158 patients in the COVID-19 positive group and 302 in the COVID-19 negative group [13, 15, 17]. The pooled results showed that the level of D-dimer was significantly higher in the COVID-19 positive group than the COVID-19 negative group (MD = 1062.51; 95% CI: 246.83 to 1878.20, *P* = 0.01; *P* < 0.00001 for heterogeneity) (Table 3). Two reported the level of creatinine, with 75 patients in the COVID-19 positive group and 196 in the COVID-19 negative group [13, 17]. The level of creatinine was significantly higher in the COVID-19 positive group than the COVID-19 negative group (MD = 0.10; 95% CI: 0.05 to 0.15, *P* = 0.0003; *P* = 1.00 for heterogeneity) (Table 3). The levels of amylase and lipase were reported by two studies, with 75 patients in the COVID-19 positive group and 196 in the COVID-19 negative group [13, 17]. Both the levels of amylase and lipase were significantly higher in the COVID-19 positive group than the COVID-19 negative group (MD = −519.03; 95% CI: −678.25 to −503.80, *P* < 0.00001; *P* = 0.38 for heterogeneity for amylase; MD = −841.95; 95% CI: −1065.08 to −618.82, *P* < 0.00001; *P* = 0.33 for heterogeneity for lipase) (Table 3). Three studies reported the level of C-reactive protein (CRP), with 158 patients in the COVID-19 positive group and 302 in the COVID-19 negative group [13, 15, 17]. The level of CRP was significantly higher in the COVID-19 positive group than the COVID-19 negative group (MD = 0.40; 95% CI: 0.16 to 0.64, *P* = 0.001; *P* < 0.00001 for heterogeneity) (Table 3). There was no significant difference in terms of lactate dehydrogenase, calcium, and procalcitonin.

### 3.5. Sensitivity Analysis

For the above analyzed pooled items which included no less than three studies, a sensitive analysis was conducted. The pooled results of the hospital stay changed with no significant difference between the two groups after the removal of Pandanaboyana et al. study (MD = −5.01; 95% CI: −12.01 to 2.00, *P* = 0.16; *P* = 0.01, *I*^2^ = 77% for heterogeneity) [11] and Dirweesh et al.'s study (MD = −5.05; 95% CI: −10.92 to 0.83, *P* = 0.09; *P* = 0.008, *I*^2^ = 79% for heterogeneity) [14]. As for leucocyte, the pooled results changed with significant difference and low heterogeneity between the two groups after the removal of Karaali's study (MD = −1.28; 95% CI: −2.43 to −0.12, *P* = 0.03; *P* = 0.21, *I*^2^ = 37% for heterogeneity) [15]. The level of CRP between the COVID-19 positive group and the COVID-19 negative group changed with no difference after the removal of Karaali's study (MD = 0.01; 95% CI: –0.01 to 0.04, P = 0.24; P<0.0001, I2=100% for heterogeneity) [15]. However, the need for surgery, the level of lymphocytes, and D-dimer between the two groups remained consistent when performing the sensitive analysis.

### 3.6. Publication Bias

**Figure 1:**
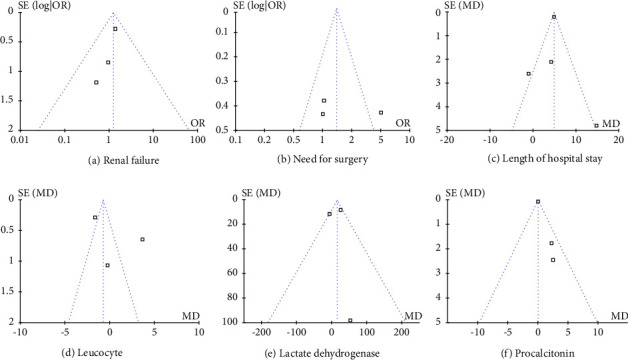
Funnel plot for pooled results. Regarding the funnel plots on pancreatic necrosis, ARDS/ALI, mechanical ventilation, ICU admission, mortality, leucocyte, platelets, and C-reactive protein, all the included studies lay inside the limits of the 95% confidence interval, showing there is no serious publication bias. As for D-dimer, the funnel plot shows included studies lay outside the limits of the 95% confidence interval. ARDS, acute respiratory distress syndrome; ALI, acute lung injury; ICU, intensive care unit.

As is shown in Figure 1, the funnel plots of pancreatic necrosis, ARDS/ALI, mechanical ventilation, ICU admission, mortality, leucocyte, platelets, and C-reactive protein showed all included studies lay inside the limits of the 95% confidence interval, indicating that there is no serious publication bias (Figures 1(a)–1(g) and Figure 1(i)). As for D-dimer, the funnel plot shows the included studies lay outside the limits of the 95% confidence interval, indicating there exists a publication bias (Figure 1(h)). The funnel plots of other pooled results are shown in Figure 1S.

## 4. Discussion

COVID-19 patients with pancreatic symptoms have caught increasing attentions. Pancreatic islet cells have been found expressing ACE2, which is the major receptor of COVID-19 on the pancreas. [6, 18]. In a series of 121 patients with COVID-19, Liu et al. [6] discovered that the risks of developing pancreatitis were much higher for patients with severe COVID-19. It is supposed that the pancreas injury may be due to the direct cytopathic effect mediated by COVID-19. Moreover, this pancreas injury may be caused by the exaggerated immune response with subsequent cytokine storm and endothelial damage triggered by COVID-19 in the clinical condition such as the Systemic Inflammatory Response Syndrome (SIRS) [7]. In the present meta-analysis, COVID-19 patients with AP were associated with a higher risk of pancreatic necrosis and SAP. This is partly in line with the study by Jin et al., in which the rate of severe condition for COVID-19 positive patients was significantly higher in patients with gastrointestinal symptoms [19].

In line with previous studies which found cardiac injury and failure were prevalent and associated with COVID-19 disease, the incidence of cardiovascular failure in COVID-19 positive patients was higher in the present study [20]. The present study showed COVID-19 positive AP patients were associated with a high incidence of persistent organ failure, which may be attributed to the higher incidence of cardiovascular failure and ALI/ARDS induced by COVID-19 infection. Moreover, COVID-19 positive AP patients were associated with high mortality rates. These results further confirmed the more severe condition and deteriorated outcomes for COVID-19 positive patients, which is in line with the findings by Akarsu et al. [21].

Regarding the treatment measures, the present study showed COVID-19 patients with AP were associated with high demanding of mechanical ventilation, but not the use of noninvasive ventilation. Furthermore, the present study found COVID-19 patients with AP were associated with a higher rates of ICU admission as well as longer hospital stay. The increased demandings of intensive care interventions were coincided with the disease severity of these patients groups with higher persistent organ failure and mortality rates.

In the present study, although COVID-19 patients with AP were not associated with a higher incidence of renal failure, COVID-19 patients with AP were associated with increased levels of creatinine. As acute kidney injury is one of the common complications of severe acute pancreatitis, this result also indicated more severe conditions for COVID-19 positive AP patients [22]. Moreover, as coagulopathy is a complex complication in SAP, the present study showed a more severe disorder of coagulation in COVID-19 positive AP patients involving platelets, prothrombin time, ATPP, and D-dimer [23]. In the present study, COVID-19 positive AP patients were associated with a higher level of CRP and lower level of amylase and lipase, which confirmed a more severe systemic inflammation condition for these groups of patients.

Because of the inherent limitations in the present study, consideration should be taken referring to these results. Firstly, the present study only included 7 studies, and the number of included studies is small. Funnel plots were only conducted on items which included no less than 3 studies. Thus, the risk of publication bias could not be ignored, although the funnel plots showed minimal publication bias. Secondly, as none of the included studies is RCT, selective bias could not be ignored. Thirdly, an indirect data acquisition method was employed while performing data collection. Finally, the included studies were carried out in different centers, from different countries, and involved with different races. Thus, variations always existed among the studies involving treatment experiences and clinical processes. These variations may lead to heterogeneity in some pooled results, introducing potential bias.

## 5. Conclusion

The present study confirmed that COVID-19 patients with AP were at a higher risk of developing SAP and were associated with increased mortality and persistent organ failure rates, especially ALI/ARDS and cardiovascular failure. Moreover, the COVID-19 infection induced a more severe systemic inflammation and coagulation disorder. The present study also indicated that intensive care supporting such as ICU admission and mechanical ventilation were largely demanded by COVID-19 patients with AP. Considering the deteriorated clinical condition and outcomes brought with COVID-19, more attention should be payed. Better designed comparative studies regarding the treatment for these groups of patients are still needed.

## Figures and Tables

**Table 1:** Baseline characteristics and demographics of patients included.

First author, year	Number		Age		Gender (male/female)		Atlanta Criteria (mild/moderate/severe)		Etiology (Gallstones/alcoholic/idiopathic/other)		BISAP score (<3/≥3)		NOS score
	NG	PG	NG	PG	NG	PG	NG	PG	NG	PG	NG	PG	
Pandanaboyana et al. 2020	1628	149	54.50 ± 18.10	59.90 ± 17.20^*∗∗*^	834/786	93/55^*∗∗*^	1244/256/100	71/42/33^*∗∗*^	696/434/93/428	60/28/13/49	NR	NR	7
Inamdar et al. 2020	157	32	52.14 ± 19.80	53.44 ± 16.60	61/96	14/18	NR	NR	53/58/33/13	5/2/22/3^*∗∗*^	86/71	20/12	8
Miró et al. 2020	162	54	61.00 (49–77)	68.00 (53–79)	91/71	39/15^*∗*^	NR	NR	0/43/0/51	0/9/0/11	138/24	35/19^*∗∗*^	7
Dirweesh et al. 2020	61	14	48.40 ± 14.10	55.20 ± 14.80	27/34	7/7	NR	NR	19/39/1/2	1/3/8/1^*∗∗*^	58/3	8/6^*∗∗*^	7
Karaali and Topal et al. 2021	106	83	52.21 ± 17.21	57.24 ± 16.34	48/58	42/41	NR	NR	61/0/0/45	42/0/0/41	91/15	56/27^*∗∗*^	7
Samanta et al. 2022	230	85	40.07 ± 11.90	41.10 ± 13.00	58/27	157/73	41/96/93	13/40/32	NR	NR	NR	NR	7
Haydar et al. 2022	34	21	58.20 ± 18.50	63.70 ± 16.80	17/17	14/7	30/4/0	14/0/7	NR	NR	NR	NR	7

CCI, Charlson comorbidity index; BISAP, bedside index of severity in acute pancreatitis; NOS, the Newcastle-Ottawa scale score; NR, not report; NRTF, not reported in this form; NG, COVID-19 negative group; PG, COVID-19 positive group. ^*∗*^Statistical difference, *P* < 0.05. ^*∗∗*^Statistical difference, *P* < 0.01.

**Table 2:** Pooled results of clinical outcomes.

Pooled result	Statistical method	Number of studies	MD/OR	95% CI	*P* value	Heterogeneity	
						*P*	*I * ^2^
Pancreatic necrosis	Fixed	4	1.65	1.13, 2.42	0.010^*∗*^	0.82	0%
Portal venous thrombosis	Fixed	2	1.02	0.35, 2.94	0.97	0.95	0%
Persistent organ failure	Random	2	6.87	2.37, 19.98	0.0004^*∗∗*^	0.12	58%
Cardiovascular failure	Fixed	2	2.92	1.66, 5.14	0.0002^*∗∗*^	0.58	0%
Renal failure	Fixed	3	1.27	0.76, 2.12	0.37	0.68	0%
ARDS/ALI	Fixed	4	3.03	2.09, 4.39	<0.00001^*∗∗*^	0.20	35%
MODS	Random	2	4.78	0.74, 30.77	0.10	0.02^*∗*^	81%
Mechanical ventilation	Fixed	3	7.36	4.19, 12.92	<0.00001^*∗∗*^	0.66	0%
Noninvasive ventilation	Random	2	2.92	0.54, 15.69	0.21	0.09	66%
Need for surgery	Random	3	1.67	0.62, 4.49	0.31	0.01^*∗*^	78%
ICU admission	Fixed	4	2.76	1.98, 3.85	<0.00001^*∗∗*^	0.47	0%
Length of hospital stay	Random	4	4.53	0.96, 8.10	0.01^*∗*^	0.02^*∗*^	69%
Mortality rate	Fixed	7	3.70	2.60, 5.25	<0.00001^*∗∗*^	0.12	40%

MD, mean difference; OR, odds ratio; CI, confidence interval; ARDS, acute respiratory distress syndrome; ALI, acute lung injury; MODS, multiple organ dysfunction syndrome; ICU, intensive care unit. ^*∗*^Statistical difference, *P* < 0.05. ^*∗∗*^Statistical difference, *P* < 0.01.

**Table 3:** Pooled results of laboratory tests.

Pooled result	Statistical method	Number of studies	MD/OR	95% CI	*P* value	Heterogeneity	
						*P*	*I * ^2^
Leucocyte (×10^9^/L)	Random	3	0.62	−3.04, 4.29	0.74	<0.00001^*∗∗*^	96%
Lymphocyte (×10^9^/L)	Random	3	−0.49	−0.74, −0.24	0.0001^*∗∗*^	0.07	62%
Platelets (×10^9^/L)	Fixed	3	13.41	4.64, 22.18	0.003^*∗*^	0.37	1%
Prothrombin time (s)	Fixed	2	0.31	0.12, 0.50	0.001^*∗∗*^	0.23	32%
APTT (s)	Fixed	2	1.95	1.30, 2.59	<0.00001^*∗∗*^	0.57	0%
D-dimer (ng/ml)	Random	3	1062.51	246.83, 1878.2	0.01^*∗*^	<0.00001^*∗∗*^	93%
Lactate dehydrogenase (IU/L)	Random	3	12.14	−13.68, 37.96	0.36	0.11	55%
Creatinine (mg/dL)	Fixed	2	0.10	0.05, 0.15	0.0003^*∗∗*^	1.00	0%
Calcium (mg/dL)	Fixed	2	0.09	−0.01, 0.20	0.08	0.22	33%
Amylase (IU/L)	Fixed	2	−591.03	−678.25, −503.80	<0.00001^*∗∗*^	0.38	0%
Lipase (IU/L)	Fixed	2	−841.95	−1065.08, −618.82	<0.00001^*∗∗*^	0.33	0%
C-reactive protein (g/L)	Random	3	0.40	0.16, 0.64	0.001^*∗∗*^	<0.00001^*∗∗*^	99%
Procalcitonin (*μ*g/L)	Fixed	3	0.08	−0.12, 0.28	0.42	0.28	21%

MD, mean difference; OR, odds ratio; CI, confidence interval; APTT, activated partial thromboplastin time. ^*∗*^Statistical difference, *P* < 0.05. ^*∗∗*^Statistical difference, *P* < 0.01.

## Data Availability

The processed data used to support the findings of this meta-analysis study are available from the corresponding author upon request.

## Abbreviations

AP: Acute pancreatitis
SAP: Severe acute pancreatitis
COVID-19: The novel coronavirus disease 2019
MODS: Multiple organs dysfunction syndrome
IPN: Infected pancreatic necrosis
NP: Necrotizing pancreatitis
CT: Computed tomography
ASA: American society of anesthesiologists
AST: Aspartate aminotransferase
BUN: Blood urea nitrogen
ICU: Intensive care unit
NOS: Newcastle-Ottawa Scale
RCTs: Randomized clinical trials
MDs: Mean differences
CIs: Confidence intervals
ORs: Odds ratios
APTT: Activated partial thromboplastin time
ARDS: Acute respiratory distress syndrome
ALI: Acute lung injury.

## Additional Points

This meta-analysis analyzed the clinical characteristics and outcomes of COVID-19 patients with acute pancreatitis. COVID-19 deteriorated the clinical condition and outcomes of acute pancreatitis. COVID-19 patients with acute pancreatitis (AP) were at a higher risk of developing pancreatic necrosis, and were associated with increased risk of persistent organ failure, especially cardiovascular failure and acute respiratory distress syndrome (ARDS) or acute lung injury (ALI). COVID-19 may deteriorate renal function with increased levels of creatinine as well as induce coagulation disorders involving platelets, prothrombin time, activated partial thromboplastin time, and D-dimer. The present study also indicated that intensive care supporting such as ICU admission and mechanical ventilation but not noninvasive ventilation were largely demanded by these patients. At last, COVID-19 patients with AP were associated with a higher mortality rates. Reporting Checklist: the present study was reported in accordance with PRISMA reporting checklist.

## Supplementary Materials

Supplementary Materials Figure 1S: Funnel plot for renal failure, need for surgery, length of hospital stay, leucocyte, lactate dehydrogenase, and procalcitonin. Regarding the funnel plots on renal failure, lactate dehydrogenase, and procalcitonin, all the included studies lay inside the limits of the 95% confidence interval, indicating there is no serious publication bias. Regarding the funnel plots on need for surgery, length of hospital stay, and leucocyte, parts of the included studies lay outside the limits of the 95% confidence interval. Supplementary Table 1: The PRISMA Checklist of the present study.
