# Supplementary material for: COVID-19 Infection Deteriorates the Clinical Condition and Outcomes of Acute Pancreatitis: A Meta-Analysis
Source: Emerg Med Int. 2022 Oct 28;2022:6823866. doi: 10.1155/2022/6823866 (PMC9635973; doi:10.1155/2022/6823866)
Supplement: Supplementary Materials — Figure 1S: Funnel plot for renal failure, need for surgery, length of hospital stay, leucocyte, lactate dehydrogenase, and procalcitonin. Regarding the funnel plots on renal failure, lactate dehydrogenase, and procalcitonin, all the included studies lay inside the limits of the 95% confidence interval, indicating there is no serious publication bias. Regarding the funnel plots on need for surgery, length of hospital stay, and leucocyte, parts of the included studies lay outside the limits of the 95% confidence interval. Supplementary Table 1: The PRISMA Checklist of the present study. [file 6823866.f1.zip › Figure 1s.pdf]

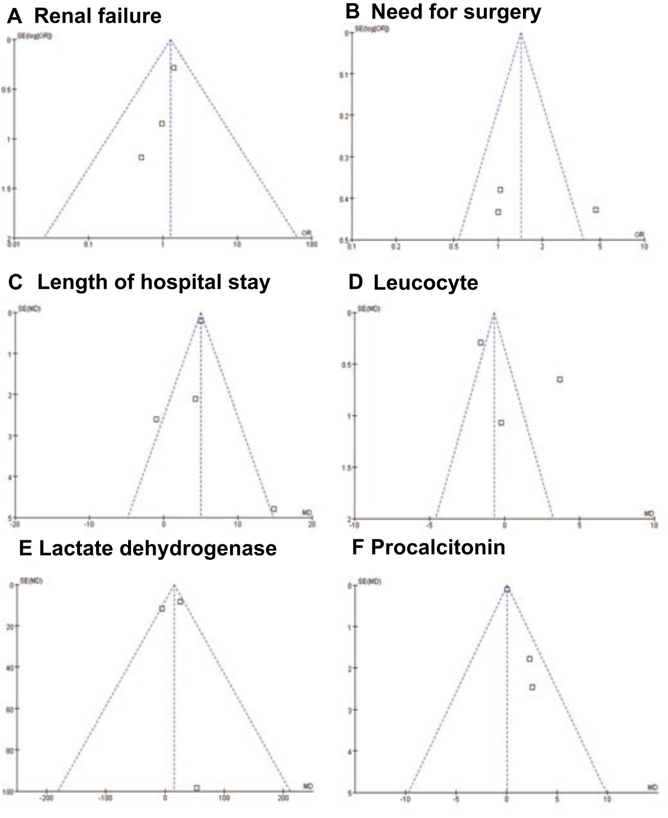
A Renal failure
SE(log[OR])
OR
0.01
0.1
1
10
100
B Need for surgery
SE(log[OR])
OR
0.1
0.2
0.5
1
2
5
10
C Length of hospital stay
SE(MD)
MD
-20
-10
0
10
20
D Leucocyte
SE(MD)
MD
-10
-5
0
5
10
E Lactate dehydrogenase
SE(MD)
MD
-200
-100
0
100
200
F Procalcitonin
SE(MD)
MD
-10
-5
0
5
10
